# Dual Plating with 4.5mm PHILOS for Managing Challenging Humerus Shaft Non-Unions: A Novel Technique

**DOI:** 10.5704/MOJ.2603.011

**Published:** 2026-03

**Authors:** P Luke, SI Sim, A Gardner, YH Ng, JH Tan

**Affiliations:** 1 Department of Orthopaedic Surgery, National University Hospital, Singapore; 2 Department of Orthopaedic Surgery, Ng Teng Fong General Hospital, Singapore

**Keywords:** humeral shaft fractures, dual plating, revision surgery, non-union, infection

## Abstract

**Introduction:**

Fixation failures of humeral shaft fractures are uncommon. When they do occur, revision fixation can be challenging. This study aims to evaluate the clinical and radiological outcomes of a novel surgical technique for managing challenging humerus midshaft fractures and non-unions, specifically assessing union and complication rates.

**Materials and methods:**

This retrospective study included all consecutive patients who underwent surgical fixation of midshaft humerus fractures using our novel dual plating technique between October 2016 and July 2019. Six patients were included. At follow-up, union was assessed using plain films.

**Results:**

Mean age was 50.8 ± 18.0 years, including 4 males and 2 females. All surgeries were revision fixations except for one primary fixation. Mean follow-up period was 6.5 ± 2.3 months. At final follow-up, 5 of 6 patients (83.3%) achieved clinical and radiographic union, with a mean time to union of 6.4 ± 2.0 months. Residual angular deformity at union averaged 1.2 ± 0.8° in the coronal plane and 2.0 ± 2.0° in the sagittal plane. Aside from one case of delayed union, no major complications were reported.

**Conclusion:**

The novel dual plating technique can be reliably executed with good results and shows promise as a surgical treatment option for non-union or implant failure. While particularly valuable in revision settings, it may be considered for challenging primary fixations. Further research is needed before recommending its widespread use in primary fracture management.

## Introduction

Humeral shaft fractures are common injuries and account for 1 to 3% of all adult fractures^1^. It is a common site for osteoporosis-related fragility fractures and a significant healthcare concern in regions with aging populations^2^. Conservative management of humerus shaft fractures with functional bracing was described by Sarmiento *et al* in 1977^3^, with excellent results reported. The high tolerance for deformity in the humeral shaft due to the large shoulder and elbow range of movement have made non-operative treatment the standard of care for up to 85% of humeral shaft fractures^4^. However, recent studies report a non-union rate of approximately 5.5% following conservative management, contrasting with Sarmiento *et al’s* findings of no non-union cases in a cohort of 51 patients^3,5,6^. Other studies have also found surgical treatment to be associated with higher fracture healing rates, fewer complications, and better functional outcomes^7^.

Surgical treatment of humeral shaft fractures has traditionally involved open plating, although intramedullary nailing for such fractures is also increasingly performed as a minimally invasive alternative to open plating^8^. The optimal mode of fixation remains controversial. Fixation failures of humeral shaft fractures are uncommon given the non-weightbearing nature of the bone and the good inherent blood supply to the humerus, with extensive vascular anastomoses in all segments of the bone. However, when such failures do occur, management with revision fixation is often challenging and there is limited literature available to guide treatment.

This case series describes a novel dual plating technique used in six patients for surgical fixation of midshaft humerus fractures. The technique was applied in various clinical scenarios, including surgical management after failed conservative treatment, and revision of failed surgical fixation. Here, we present the surgical approach and outcomes of this fixation strategy.

## Materials and Methods

This retrospective study includes all consecutive patients who underwent surgical fixation of midshaft humerus fractures using our dual plating technique between October 2016 and July 2019. Institutional Review Board approval was obtained (IRB number 2019/01240). A total of six patients were included.

All surgeries were performed by two senior authors of the study. General anaesthesia was used for all patients. Patients were positioned supine on a radiolucent MARS operating table [TRUMPF Medizin Systeme GmbH, Germany], which facilitates intra-operative imaging by eliminating obstruction by radio-opaque metallic siderails. A towel roll was placed in the interscapular region to elevate operative shoulder, improving on-table shoulder mobility and facilitating access to both the anteromedial and lateral aspects of the humerus for easier lateral plate placement. No bone grafting was used in all cases.

The anterolateral approach to the humerus was utilised for anterior plating. The skin incision was performed along the lateral border of the biceps, centred on the fracture site. The biceps was retracted medially and the intermuscular plane between brachialis and triceps proximally or between brachialis and brachioradialis distally was developed. The radial nerve was identified and protected. The lateral border of the brachialis was released off the anterolateral aspect of the humerus and retracted medially to expose the shaft of the humerus. Anterior plating was performed with the plate placed on the anteromedial aspect of the humeral shaft. Lateral plating was performed in a minimally invasive fashion with a deltoid splitting MacKenzie approach utilised proximally and the already developed intermuscular plane between brachialis and brachioradialis distally^9^.

The anterolateral approach provided us with the proximal and distal visualisation needed for fracture reduction, and biological augmentation where necessary. Preliminary fixation with an anterior plate facilitated subsequent long lateral plate placement. Our fixation construct of choice was a 3.5mm locking compression plate (LCP) applied anteriorly, and a long pre-contoured proximal humerus internal locking system plate (PHILOS) plate [Synthes, Switzerland] or 4.5mm LCP applied laterally. Adjustments to the pre-contoured plate were commonly made prior to final insertion to improve the matching to the bone contours, particularly to accommodate the flare of the distal humerus and to avoid plate prominence proximally, which could lead to subacromial impingement. The long PHILOS plate was used to provide a long lever arm fixation. Together with the anterior shorter plate, this allows for rigid fixation of the fracture site, while resisting the large torsional and bending forces at the shoulder and elbow regions.

Post-operatively, all patients were allowed immediate unprotected range of movement exercises of both the shoulder and elbow. Strengthening and weight bearing exercises were started once there was clinical and radiographic evidence of fracture healing, with a focus on deltoid strengthening exercises. At follow-up, union was assessed using plain radiographs.

## Results

A total of 6 patients underwent surgical fixation using our novel dual plating technique during the study period. The mean age of patients in the study was 50.8 ± 18.0 (21 -71) years. Four patients were male, and two patients were female. All the male patients were smokers. Both female patients were non-smokers (Table I). Mechanism of injury was secondary to road traffic accidents in three patients, falls in two patients and blunt trauma in one patient. All fractures in this series were closed fractures. Radial nerve palsy was not present for any of the patients pre-operatively.

**Table I:** Patient demographics and clinical characteristics.

Age (years)	Sex			Smoking Status	Dual plating used as primary or revision surgery	Side of fracture	AO/OTA Classification	Indication Time from injury to dual plating surgery (months)			Duration of follow-up (months)		Outcome
49	Female			Non-smoker	Revision	Right	12-A3	Oligotrophic 10 non-union			11		Union
57	Male			Smoker	Revision	Left	12-A3	Hypertrophic 25 non-union			4		Union
65	Male			Smoker	Primary^1^	Left	12-A1	Oligotrophic 7 non-union			6		Union
21	Male			Smoker	Revision	Right	- ^2^	Pseudoarthrosis 32			6		Union
71	Female			Non-smoker	Revision	Right	12-A3	Traumatic 1 peri-implant fracture			6		Union
42	Male			Smoker	Revision	Left	- ^2^	Septic non-union 102			6		Delayed union – lost to follow-up

^1^ Dual plating was performed as a primary surgery after failure of conservative treatment. ^2^Initial post-injury radiographs were obtained in a different country and were unavailable for review by the authors.

All dual plating surgeries were performed as revision fixations except for a single case, where it was used for primary fixation. The indications for surgery included early implant failure in one patient and non-union in five patients. The non-union cases comprised a spectrum of types: oligotrophic non-union (2 patients), hypertrophic non-union (1 patient), pseudoarthrosis (1 patient), and septic non-union (1 patient).

The mean follow-up period was 6.5 ± 2.3 (4 – 11) months post-operatively. At the end of their respective follow-up periods, 5 of 6 patients (83.3%) achieved clinical and radiographic union. The single patient who did not achieve union was lost to follow-up at six months post-operatively, as he was migrating overseas. On the last clinical review, he was pain-free and had full function of his arm. Plain films showed callus formation, but radiographic union had not been achieved yet.

The mean time to union was 6.4 ± 2.0 (4 – 10) months. Residual angular deformity at union was a mean of 1.2 ± 0.8 (0 – 2) degree in the coronal plane and 2.0 ± 2.0 (0 – 4) degree in the sagittal plane. Other than the single case of delayed union, there were no other complications in our series, such as infection, nerve palsy or prominent hardware. The single case of delayed union occurred in a patient whose initial fixation was complicated by septic non-union. He underwent removal of implants and ring external fixator stabilisation which was again complicated by non-union and eventually underwent fixation with the dual plating technique.

## Discussion

**Fig. 1:**
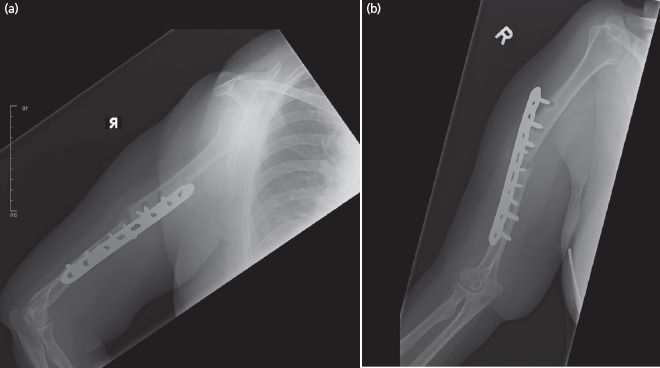
Radiographs taken at nine months post-primary surgery. En masse proximal screw pull-out of previous locking plate fixation (AP and lateral view). The most proximal screw was confirmed to be intracortical. The second and third screws were unicortical.

Surgical fixation of humerus midshaft diaphyseal fractures is commonly performed with intramedullary nailing or open reduction and internal fixation with a large fragment plate with at least 2 to 3 screws per segment depending on bone quality and fracture configuration^8^. The use of a locking plate further improves fixation strength in the setting of osteoporotic bone. However, these constructs are associated with their unique set of pitfalls. In particular, the high stiffness of these locked plates with angular stability of the screws can cause them to fail dramatically with en masse screw pull-out from bone which can be very difficult to salvage. This mode of failure was observed in two patients in this series, both of whom had previously undergone internal fixation with a locking plate, which later failed due to en masse pull-out of the proximal screws (Fig. 1).

It is crucial to minimise the risk of another failure in the setting of any revision fracture fixation, and most surgeons would prefer to err on the side of “overdoing” the fixation. There is minimal guidance on strategies to manage this in the literature. Management options that have been described include external fixation, intramedullary nailing with decortication of the non-union site and plating with biological augmentation^10^. However, these methods are not without limitations. The use of external fixators in the humerus is usually less well tolerated as compared to the lower limbs, and the limited safe corridors for pin and wire placement make a stable fixator construct more difficult to achieve. Intramedullary devices in the upper limb may not achieve the fracture site compression necessary for union especially in the setting of a failed fixation. Each of these methods is also associated with its own set of complications, including pin site infections for external fixators and a significant rate of shoulder pain and dysfunction with intramedullary nailing^11,12^.

The concept of dual plating is commonly employed in distal humerus metaphyseal fractures, where it is essential to resist the large torsional and bending forces at the periarticular region of elbow. The use of dual plating in the setting of humerus diaphyseal fractures is less well described. Biomechanical studies have demonstrated mechanical superiority of dual plating of humeral shaft fractures over a single plate construct^13^. Finite element analysis performed by Kosmopoulos *et al* showed that an orthogonal plate placement confers greater compressional and torsional stiffness as compared to side-by-side plate configuration^14^. Previous clinical studies have described the use of dual plating in distal third diaphyseal fractures of the humerus^15,16^. Lee *et al* described usage of a 3.5mm reconstruction plate as a “reduction plate” applied anteriorly, followed by definitive fixation with a 4.5mm locking compression plate applied laterally^15^. The authors described the benefits of this fixation to be a smaller skin incision and soft tissue stripping due to the shorter required working length required secondary to the increased mechanical rigidity of the dual plate construct. Other studies have described the use of dual plating for proximal humeral fractures, including the technique published by Choi *et al,* with a combination of a proximal humeral plate (PHILOS) and a standard locking compression plate^17,18^. However, there are few in vivo studies that have investigated dual plating of midshaft fractures of the humerus. A recent such study by Seo *et al* found that dual plating of these fractures had satisfactory union rates with no increase in complications or surgical time compared to single plating^19^. Rubel *et al* also presented a case series of dual plating for humerus shaft non-unions^20^. However, there were no cases of usage of the dual plating techniques for revision surgery in the series by Seo *et al.* Furthermore, the surgical techniques employed in both studies differ significantly from our own, as described below.

In a recent study by Seo *et al*, a short 3.5mm locking compression plate was applied to the anteromedially and subsequently, a 3.5mm locking compression plate was applied anterolaterally^19^. Rubel *et al* opted for placement of a 4.5mm LCDCP plate on the posterior aspect and a short 3.5mm reconstruction plate on the lateral aspect of the humerus^20^. Our preference for plate placement is anteromedial and direct lateral. The anteromedial surface of the humerus provides a convenient bony ridge for plate placement, and it can be easily accessed through the anterolateral approach to the humerus, which is familiar to most orthopaedic surgeons. We preferred this approach as compared to the standard anterior approach as it allowed us greater exposure distally as opposed to the standard anterior approach, which is limited distally by the lateral antebrachial cutaneous nerve. This is the first plate that we apply, as it also acts as a provisional fixation device to ease placement of the long spanning lateral plate. The technique of reduction plating was previously described by Archdeacon *et al*^21^. We prefer to use 3.5mm plates rather than large fragment plates for anterior plating in our dual plate fixation constructs to reduce the size of screw holes within bone that is already deficient. Smaller screw holes also reduce the risk of screw hole convergence and would make it easier to “miss-a-screw” when inserting screws for the lateral plate. The mechanical strength of the fixation is derived from the orthogonal plate configuration rather than screw size in such a construct. Distal fixation anteriorly is challenging due to the presence of multiple neurovascular structures running anteriorly over the distal humerus, including the lateral antebrachial cutaneous nerve, brachial artery and median nerve. Any additional distal fixation should, therefore, be done through the lateral plate subsequently. Proximally, fixations can extend all the way to the surgical neck of the humerus, with careful subperiosteal elevation of the distal pectoralis major tendon insertion as required. The second plate that we apply is a long PHILOS plate, inserted laterally in a minimally invasive fashion, utilising a MacKenzie deltoid-splitting approach proximally and the already developed interval from the anterolateral approach, between the brachialis and brachioradialis distally^9^. The proximal exposure usually does not need to be larger than 5cm for placement of head screws through the pre-contoured locking plate. The axillary nerve is palpated on the deep surface of the deltoid and protected. The use of a long PHILOS plates or 4.5mm LCP, instead of the recon plates used by Rubel *et al*^20^, also provides a more mechanically robust implant that is less susceptible to failure. An important consideration for placement of the lateral plate is the need to strip a small portion of the deltoid insertion from the deltoid tuberosity to allow the proximal to distal sliding of the long plate. This can either be done using a blunt elevator through the proximal deltoid split incision or alternatively through the anterolateral humerus incision under direct vision. Distal exposure between the brachialis and brachioradialis gives us a direct end-on view of the distal end of the plate as it curves about the lateral flare of the distal humerus. The radial nerve should be visualised and protected as it travels from the posterior compartment of the arm to the anterior compartment and through the brachialis-brachioradialis interval, prior to distal screw fixation.

**Fig. 2:**
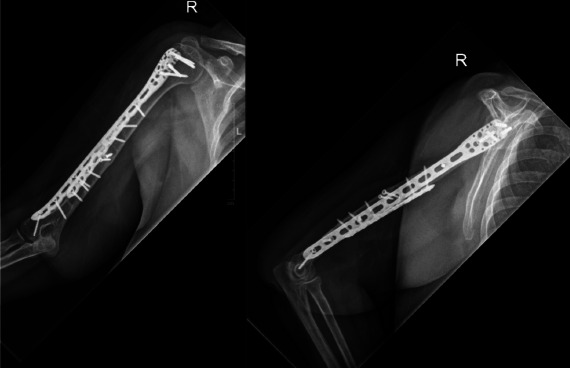
Successful union achieved with dual plating for a humeral non-union following a failed intramedullary nailing (AP and lateral view).

In most of our cases, we employed our dual plating technique for management of non-union following failed surgical fixation. Our rationale for applying a dual plate construct was to achieve increased mechanical strength of the construct, with greater resistance to deforming forces via biplanar fixation. The use of two plates also reduced the stresses on each plate, allowing us to place screws of the anterior plate closer to the fracture site to achieve greater rigidity, while minimising the risk of plate failure. The enhanced strength and rigidity of such a dual fixation construct (Fig. 2) optimises the mechanical environment for fracture healing to occur and allows rapid progression of post-operative rehabilitation.

The difference in lengths of the short anterior plate and the long lateral plate conferred numerous benefits. We were able to achieve good screw purchase by avoiding areas of bone loss or bone defects due to the large selection of screw holes available at different portions of the humerus. The difference in plate length also gave us the ability to place screws without encountering difficulties of screws of the lateral plate impinging on screws of the anterior plate. This is a distinct benefit compared to the use of two similarly sized plates as employed by Seo *et al*^19^.

Disadvantages of our dual plating technique as compared to the technique described by Seo *et al* are mainly the additional skin incisions and soft tissue stripping cause by the insertion of the long lateral plate^19^. Although cadaveric studies have shown that the main nutrient artery to the humerus shaft enters at the mid-diaphyseal region through the anteromedial face of the bone, the vast anastomoses arising both proximally and distally make devascularization of the bone unlikely^22^. The additional soft tissue injury is therefore unlikely to significantly impair bone healing and has not been problematic in our experience with this technique. Another potential disadvantage is the need to strip a small portion of the deltoid off its distal insertion to accommodate the long lateral plate, which may cause initial deltoid weakness and delay rehabilitation. However, previous studies have shown excellent long-term functional outcomes with this approach to lateral plate insertion^23^.

In this case series, we had an 83.3% union rate, with minimal deformity post-operatively. Using the above techniques, we successfully treated midshaft diaphyseal humerus fractures in the settings of non-unions secondary to failed conservative management, non-union following surgical management, early implant failure, and septic non-union. Our single case of that did not achieve union at follow-up after revision surgery was a case that initially presented with a septic non-union. Previous studies have shown that treatment of non-union is challenging, with union rates as low as 66% even with two-stage surgery and persistent infection in up to 60% of cases^24^. Hence, our novel technique may be considered as a salvage procedure if other techniques such as standard plating or external fixation have been unsuccessful.

The limitations of this study are the relatively small sample size and lack of control group inherent to the study design. However, it provides information on a novel technique of surgical fixation of challenging midshaft humerus fractures and the results of this technique. To our knowledge, it is the only study describing this technique of dual plating for midshaft humerus fractures that has employed the technique in a spectrum of cases including non-union secondary to failed conservative management, revision surgery for non-union following surgical management, early implant failure and septic non-union.

## Conclusion

The novel dual plating technique for diaphyseal humerus fractures can be reliably executed with good results and shows promise as a surgical option for managing non-union or implant failure. However, given the limited number of primary cases in our study, further research is needed before recommending its widespread use in primary fracture management.
